# Small-molecule therapies for pediatric inflammatory bowel disease: toward precision medicine

**DOI:** 10.1007/s12519-025-01001-6

**Published:** 2025-12-06

**Authors:** Ying Chen, Yang Wang, Jing Guo, Ling-Fen Xu, Xu Teng

**Affiliations:** Department of Pediatric Gastroenterology, Shengjing Hospital of China Medical University, No. 36 Sanhao Street, Heping District, Shenyang, 110004 China

**Keywords:** Janus kinase inhibitor, Pediatric inflammatory bowel disease, Precision medicine, Small-molecule therapy, Sphingosine-1-phosphate modulator

## Abstract

**Background:**

Pediatric inflammatory bowel disease (pIBD) often begins early in life, progresses rapidly, and is associated with impaired growth and delayed development. These challenges demand treatment strategies that address both intestinal inflammation and the broader developmental needs of children.

**Data sources:**

This review summarizes current advances in small-molecule therapies for pIBD based on published clinical trials, real-world studies, and mechanistic investigations retrieved from PubMed and clinical trial registries. Special emphasis is placed on Janus kinase (JAK) inhibitors and sphingosine-1-phosphate (S1P) modulators, which represent the main translational research focus in pediatric IBD.

**Results:**

JAK inhibitors such as tofacitinib and upadacitinib have demonstrated promising efficacy in pediatric patients with refractory disease, although their use remains off-label worldwide. Long-term safety concerns persist, including infection risk, developmental effects, and potential risks of malignancy or major adverse cardiovascular events. S1P modulators such as ozanimod are under clinical evaluation in children, but robust long-term data are still lacking. Emerging technologies such as single-cell and spatial profiling have begun to reveal age-dependent remodeling of gut immune architecture, emphasizing the importance of developmentally informed therapeutic approaches.

**Conclusions:**

Small-molecule therapies offer a promising and mechanistically precise direction for the management of pIBD. Future progress will depend on age-specific clinical trials, physiologically based pharmacokinetic modeling, and biomarker discovery through integrated multiomics. Collaborative multicenter research is essential to optimize the safety and efficacy of these agents in children.

**Graphical abstract:**

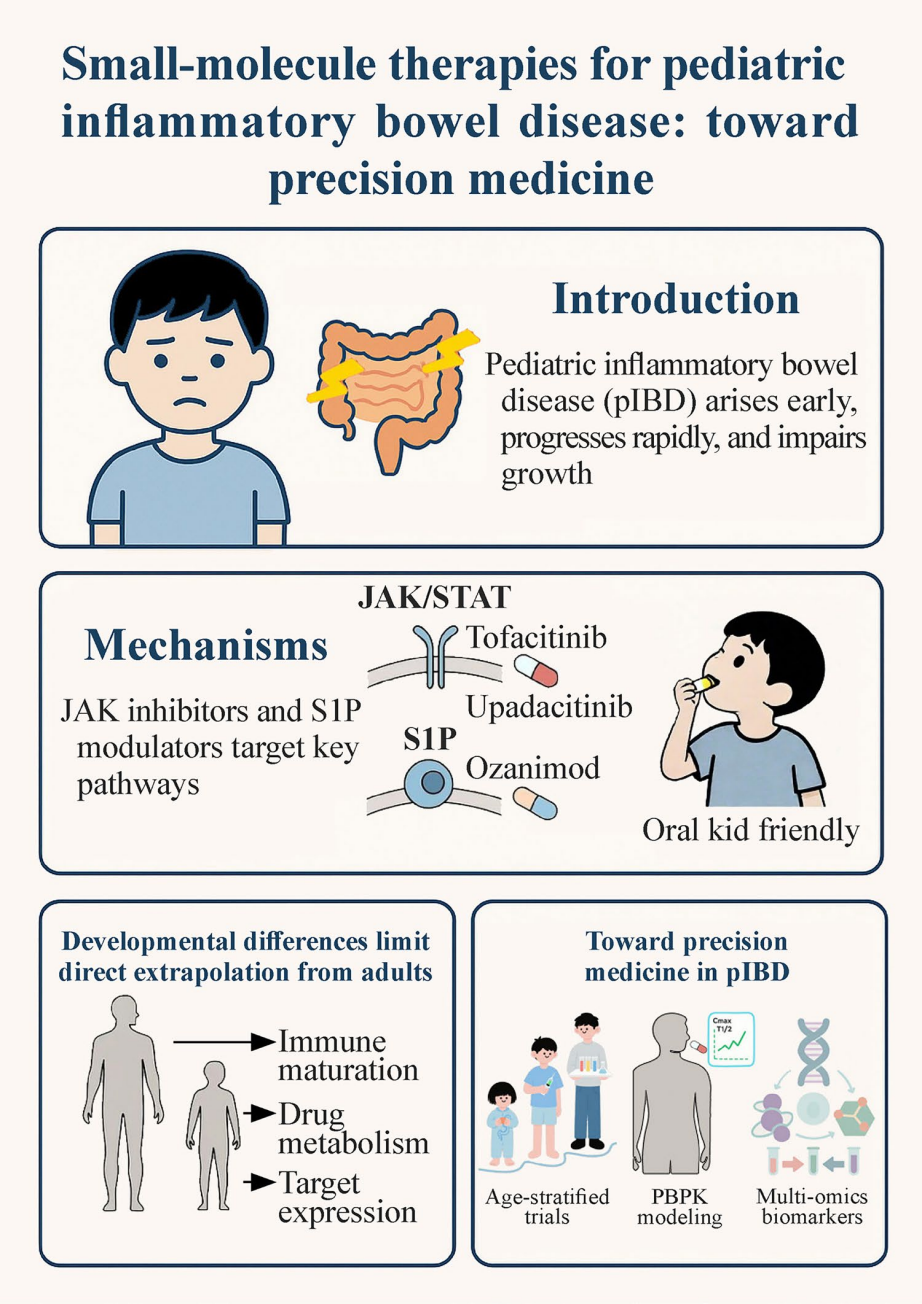

## Introduction

Pediatric inflammatory bowel disease (pIBD), which encompasses Crohn’s disease (CD) and ulcerative colitis (UC), has steadily increased in incidence over the past 25 years, making it a prominent chronic inflammatory condition in pediatric gastroenterology [1]. Unlike adult-onset IBD, pIBD tends to present with a more severe clinical phenotype, faster disease progression, and greater complications, such as growth impairment and delayed puberty [2, 3]. Therefore, treatment strategies must focus not only on controlling intestinal inflammation but also on supporting the overall growth and development of children.

Current therapeutic approaches for pIBD typically follow evidence-based clinical guidelines and involve medications (such as 5-aminosalicylic acid agents, corticosteroids, and immunomodulators) and biologic therapies (such as anti-tumor necrosis factor drugs). However, the relative lack of pediatric-specific clinical trials, along with the substantial differences in immune system maturation, pharmacokinetics, and therapeutic responses between children and adults, means that treatment regimens often need to be individualized on the basis of clinical judgment. While these therapies can manage symptoms and induce remission for many patients, their use in children has significant limitations. For example, corticosteroids may hinder growth and bone metabolism, immunosuppressive agents increase the risk of infections, and biologics can lead to secondary loss of response, the development of anti-drug antibodies, and issues with medication adherence due to injection-related discomfort [4, 5].

In recent years, small-molecule targeted therapies, including oral administration, favorable tissue penetration, and well-characterized mechanisms of action, have gained increasing attention in adult IBD treatment because of their benefits. These benefits have generated growing interest in pediatric applications, particularly since they improve treatment accessibility, enhance medication adherence, and avoid drawbacks associated with parenteral therapy. Nevertheless, pediatric-specific evidence remains scarce, and critical questions regarding indications, optimal dosing strategies, and long-term safety remain unanswered. A recent 2025 review summarized the off-label use of advanced therapies for pIBD, including small molecules such as tofacitinib and upadacitinib, highlighting promising outcomes but underscoring the urgent need for pediatric-specific data [6]. Building on this background, the present article provides a pediatric-centered focus on small-molecule therapies, incorporating the latest clinical and real-world evidence. We emphasize developmental and practical considerations that remain insufficiently addressed. Our goal is to advance the understanding of how small-molecule therapies can be optimally integrated into pIBD management. We aim to contribute to the development of individualized, evidence-based, and developmentally appropriate therapeutic approaches for children with IBD.

In recent years, small-molecule targeted therapies, such as oral administration, good tissue penetration, and well-understood mechanisms of action, have garnered increasing interest in adult IBD treatment because of their benefits. These advantages have sparked growing interest in applying these therapies in pediatric patients, especially since they may improve treatment accessibility, enhance medication adherence, and avoid issues associated with parenteral administration. However, pediatric-specific evidence remains limited, and key questions regarding appropriate indications, optimal dosing strategies, and long-term safety profiles have not been fully addressed in this population. This review aims to systematically examine small-molecule therapies for pIBD and explore their mechanisms of action, clinical efficacy, and safety considerations. We also highlight critical knowledge gaps and suggest future research directions within the framework of developmental precision medicine. Compared with previous reviews that have focused mainly on adult IBD patients, this article provides a pediatric-focused approach. It incorporates the latest pediatric clinical and real-world data and emphasizes developmental and practical issues that remain insufficiently addressed. Our goal is to help optimize treatment strategies for pIBD and contribute to the development of individualized, evidence-based, and developmentally appropriate therapeutic approaches.

## Mechanisms and clinical translation of small-molecule drugs in inflammatory bowel disease

### Characteristics of small-molecule drugs

Small-molecule drugs are chemically synthesized compounds with molecular weights typically < 1 kDa and possess flexible three-dimensional structures that facilitate cellular uptake [7, 8]. Compared with larger biological agents, such as monoclonal antibodies, small-molecule drugs offer significant pharmacokinetic advantages, such as efficient transmembrane permeability and oral bioavailability. These features enable small-molecule drugs to modulate intracellular inflammatory signaling pathways, such as those involving Janus kinase and phosphodiesterase 4, or to target membrane-associated G protein-coupled receptors, including sphingosine-1-phosphate (S1P) receptors, ultimately regulating immune responses and suppressing intestinal inflammation [9].

In pIBD, small-molecule agents offer several clinically relevant benefits. First, their oral route of administration is particularly advantageous for pediatric patients, reducing injection-related discomfort and potentially improving treatment adherence. Second, their relatively low immunogenicity minimizes the risk of anti-drug antibody formation, thus lowering the incidence of both primary and secondary loss of therapeutic efficacy [10]. Although the short half-life of most small-molecule drugs typically requires once- or twice-daily dosing, their rapid onset of action ensures quick symptom relief and effective inflammation control, helping to prevent severe disease complications [11].

However, there are pharmacological limitations to consider. Oral small-molecule drugs may have suboptimal bioavailability and undergo hepatic and renal metabolism, with potential drug‒drug interactions that could impact both efficacy and safety [9]. Despite these challenges, the overall convenience, accessibility, and pharmacodynamic precision of small-molecule therapies make them promising and practical treatment options for pIBD, especially when tailored to the developmental needs of pediatric patients.

### Core pathophysiological mechanisms of inflammatory bowel disease and targeted small-molecule drugs

The core pathophysiological mechanism of IBD involves the disruption of intestinal mucosal homeostasis driven by chronic inflammatory cycles. These cycles begin with epithelial barrier dysfunction, immune dysregulation, and changes in the gut microbiota [12, 13]. Within this framework, the Janus kinase (JAK)/signal transducer and activator of transcription (STAT) pathway and the S1P axis play key roles in regulating both the propagation of inflammation and the repair of the epithelial barrier. Recent advancements in small-molecule drugs targeting these pathways have improved IBD management in adults and show promise for pediatric populations.

**Fig. 1:**
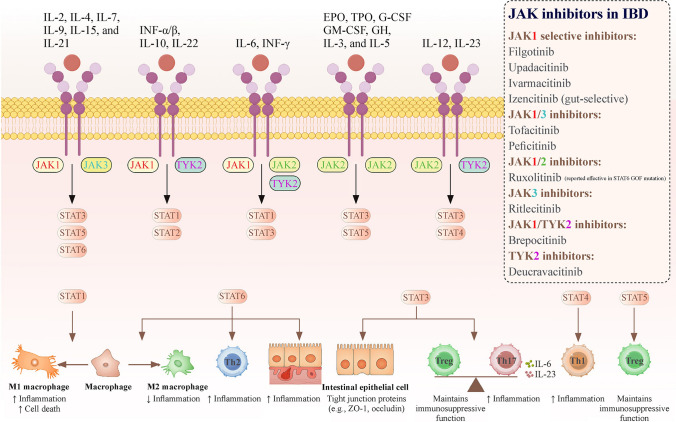
Mechanisms and therapeutic targets of JAK inhibitors in IBD. The engagement of cytokine receptors activates JAK family members (JAK1, JAK2, JAK3, and TYK2), leading to STAT phosphorylation and nuclear translocation. This pathway regulates key immune processes in IBD, including M1/M2 macrophage polarization, intestinal epithelial barrier integrity, and the balance between Treg and Th17 differentiation. Several STAT proteins (e.g., STAT3 and STAT6) play dual roles in both protective and pro-inflammatory processes, underscoring the complexity of pathway selectivity. Importantly, gain-of-function mutations in STAT family members, such as ruxolitinib, have been reported to respond to JAK inhibition, highlighting the clinical relevance of these pathways. The right panel lists selective JAK inhibitors categorized by their JAK target selectivity (JAK1-selective, JAK1/3, JAK1/2, JAK3, JAK1/TYK2, and TYK2). Some agents are approved for adult IBD (e.g., tofacitinib and upadacitinib), whereas others remain in clinical development (e.g., izencitinib, brepocitinib, and deucravacitinib). Notably, all JAK inhibitors are currently off-label in pediatric IBD patients. *JAK* Janus kinase, *STAT* signal transducer and activator of transcription, *IBD* inflammatory bowel disease, *TYK2* tyrosine kinase 2, *Treg* regulatory T cells, *IL* interleukin, *INF* interferon, *EPO* erythropoietin, *TPO* thrombopoietin, *G-CSF* granulocyte-colony-stimulating factor, *GM-CSF* granulocyte macrophage-colony-stimulating factor, *GH* growth hormone

The roles of JAK/STAT signaling in IBD are highly context dependent (Fig. 1). STAT1 predominantly drives pro-inflammatory activity, whereas STAT3 has dual functions. In epithelial cells, STAT3 promotes proliferation and barrier integrity, whereas in immune cells, it facilitates Th17 differentiation and mucosal inflammation [14–19]. Likewise, STAT6 plays dual roles. It contributes to anti-inflammatory macrophage polarization and mucosal repair but also drives type 2 inflammation through interleukin (IL)-4/IL-13 signaling. STAT6 has been implicated in the disruption of epithelial barrier function, thereby aggravating colitis [20, 21]. Other STAT family members are also involved: STAT2 is involved in type I interferon signaling, but its role in IBD remains poorly defined; STAT4 promotes IL-12-driven Th1 polarization, which is particularly relevant in CD; and STAT5 supports regulatory T-cell development and immune tolerance, with impaired activity contributing to chronic inflammation [22–24]. Variants in JAK2 and STAT3 have been linked to IBD susceptibility [1], underscoring the importance of pathway selectivity in therapeutic development. Clinically, the use of JAK inhibitors has progressed from broad-spectrum compounds to agents with greater selectivity or gut-restricted activity. Tofacitinib, the first approved JAK inhibitor, showed clear efficacy in UC but limited benefit in CD. In contrast, upadacitinib produced favorable outcomes in both UC and CD patients, highlighting the therapeutic value of JAK1 selectivity. More recent candidates—such as izencitinib, a gut-restricted inhibitor, and brepocitinib, a dual JAK1/tyrosine kinase 2 (TYK2) inhibitor—are being developed to optimize efficacy while reducing systemic toxicity. These advances may expand treatment options for pIBD, although long-term safety data remain limited [25–28]. For children, determining age-appropriate dosing, supporting adherence to oral therapy, and implementing structured long-term safety monitoring remain central challenges.

**Fig. 2:**
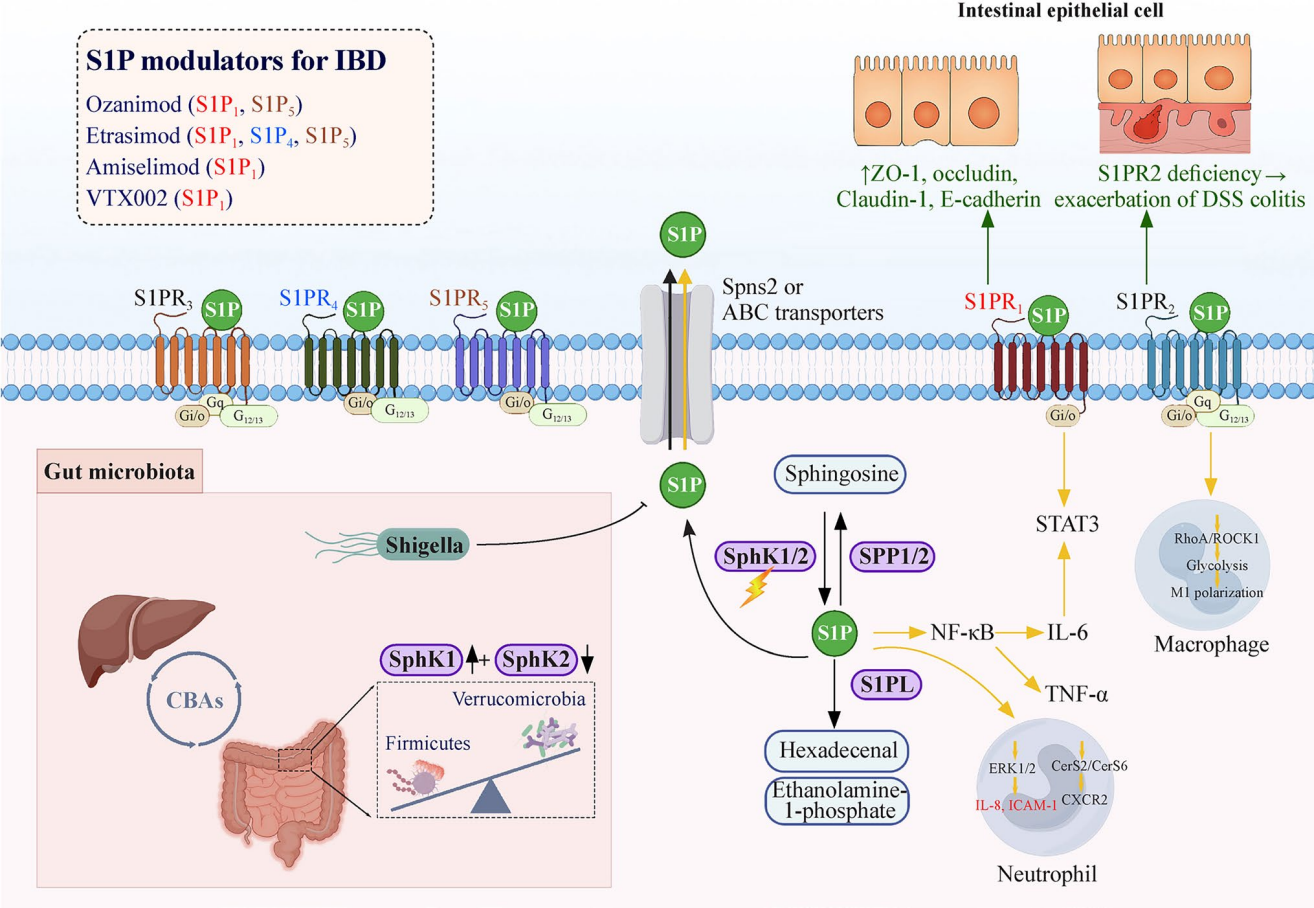
S1P signaling axis and modulators involved in the pathogenesis and treatment of IBD. Sphingosine-1-phosphate (S1P) is generated via sphingosine kinase (SphK1/2) and interacts with five S1P receptors (S1PR1–5), mediating diverse immune and epithelial responses. In the gut, S1P signaling regulates epithelial tight junction proteins, macrophage polarization, neutrophil recruitment, and inflammatory cytokine production (e.g., IL-6 and TNF-α). The gut microbiota and bile acids modulate S1P metabolism. The upper left panel summarizes S1P receptor modulators under clinical development for the treatment of IBD. *CBAs* conjugated bile acids, *IBD* inflammatory bowel disease, *DSS* dextran sulfate sodium, *NF-ĸB* nuclear factor-ĸB, *IL* interleukin, *TNF-α* tumor necrosis factor-α, *STAT* signal transducer and activator of transcription, *ERK1/2* extracellular signal-regulated kinase 1/2, *ICAM-1* intercellular adhesion molecule 1, *ROCK1* Rho-associated coiled-coil kinase 1

The S1P pathway plays a dual role in IBD, contributing to both inflammatory and reparative processes (Fig. 2). Dysregulated sphingosine kinase activity results in S1P accumulation, which enhances lymphocyte trafficking and fuels inflammation through pathways such as S1PR1/STAT3 and S1PR2-mediated macrophage activation [29–33]. In contrast, S1P receptor signaling can reinforce epithelial barrier integrity, limit apoptosis, and promote regeneration [34]. Disturbances in the sphingosine kinase 1 (SphK1)/SphK2 balance have also been linked to shifts in the gut microbiota and bile acid metabolism, creating a feed-forward loop that sustains intestinal inflammation [35]. The coexistence of pro-inflammatory and reparative actions makes the S1P axis an attractive therapeutic target. Therefore, drug development has moved beyond the nonselective modulator fingolimod, whose cardiovascular and hepatic toxicity has limited its use, toward more selective agents. Ozanimod and etrasimod have shown favorable efficacy and safety in UC and CD, while strategies targeting upstream kinases such as SphK1 or downstream degradative enzymes are actively being investigated. However, in pIBD, translation remains at an early stage. Ongoing phase II/III trials of ozanimod and etrasimod are expected to inform optimal dosing and long-term safety, particularly in relation to myelination and immune maturation. The oral route of administration provides a practical advantage for adherence in children, but individualized dosing and careful developmental monitoring will be essential as these agents move into pediatric practice.

### From adults to pediatrics: challenges and opportunities in translational medicine

UC in adults and children shares significant similarities at the gene expression level, providing a foundation for translating therapeutic strategies to pediatric patients [36]. However, the immune system in pIBD patients is not simply a “smaller version” of the adult system; rather, it exists in a developmentally active and highly adaptable state. As a result, applying adult treatment regimens directly to children may present inherent risks. Single-cell studies have shown that in the mucosa of pIBD patients, immune lymphoid cells undergo reprogramming from group 3 innate lymphoid cells to group 1 innate lymphoid cells, with transcriptional signatures characteristic of developmental stages, including Krüppel-like factor 2 and L-selectin. These findings suggest that the immune microenvironment in children has distinct features, both in terms of cellular composition and functional regulation [37]. This discovery offers a new perspective on the role of key signaling pathways in pIBD.

In the developing gut, STAT3 is more active in stem cells, where it plays a critical role in maintaining tissue homeostasis through the Wingless/integrated/β-catenin signaling pathway [38]. Furthermore, S1P-mediated regulatory T-cell homing is more pronounced before puberty, highlighting its important role in early immune regulation [39]. Notably, JAK inhibitors may affect skeletal development, whereas S1P modulators could interfere with myelin formation, emphasizing the need to consider developmental factors when evaluating drug safety. Future research should integrate single-cell genomics with developmental data to create a pediatric-specific signaling map, which could support personalized dosing strategies and the identification of new biomarkers.

While mechanistic and developmental insights are essential, translating these therapies into pediatric practice also requires addressing practical challenges that differ from those in adult care. These include determining age-appropriate dosing guided by developmental pharmacokinetics; supporting adherence to oral regimens in school-age children and adolescents with attention to formulation suitability and caregiver involvement; and implementing long-term safety monitoring that accounts for growth, vaccination schedules, and pubertal transition. These pediatric-specific considerations are discussed in greater detail in the next section.

## Application and challenges of small-molecule drugs in pediatric patients with inflammatory bowel disease

### Core drug types and clinical experience

In the management of pIBD, off-label medication use has become routine, especially for patients with moderate to severe disease or those who do not respond adequately to conventional treatments [40]. Owing to the lengthy development timelines, ethical challenges, and limited sample sizes, many small-molecule immunomodulators approved for adult use are still being studied in pediatric populations. The clinical application of these drugs is supported by real-world evidence, case reports, or small-scale studies, highlighting both the urgent clinical need and the difficulties posed by limited data and the complexity of safety evaluations (Table 1). Recently, the use of oral small molecules such as JAK inhibitors and S1P modulators has significantly changed the therapeutic landscape for adult IBD, and these drugs are now increasingly incorporated into pediatric treatment protocols as either adjuncts or alternative options.

**Table 1:** Real-world evidence on small-molecule therapies in pediatric inflammatory bowel disease

Study (country, year)	Drug	Design/population	Dosing	Key outcomes
International, 2025 [41]	Tofacitinib	Multicenter retrospective; *n* = 101; mean age 12.8 y; UC	Mostly 10 mg BID; some lower	Week 8: remission 16%, response 30%; week 24: remission 23%
Canada, 2021 [42]	Tofacitinib	Case report; 13-y-old girl; steroid-dependent UC; anti-TNF failure	5 mg BID	Rapid remission, steroid withdrawal; sustained 9 mon
USA, 2022 [43]	Tofacitinib	Retrospective; *n* = 11; mean age 17 y; UC; anti-TNF failure	10 mg BID; some TID	Colectomy-free survival day 90: 8/11; long-term bowel preservation 6/11
Canada, 2022 [44]	Tofacitinib	Case report; 14.5-y-old boy; severe multi-refractory UC	10 mg BID → 5 mg BID (week 9)	Complete remission at 9 wk; sustained 6 mon
Japan, 2022 [45]	Tofacitinib	Case report; 12-y-old boy; refractory UC	10–20 mg/d (dose escalation)	Clinical remission 3 mon; endoscopic remission 9 mon
Japan, 2023 [46]	Tofacitinib	Case report; 9-y-old boy; steroid-dependent UC; biologic failures	5 mg BID	PUCAI remission at 16 wk; sustained 52 wk
USA, 2021 [47]	Tofacitinib	Single-center retrospective; *n* = 21; median age 18.4 y; UC/IBD-U/CD; anti-TNF failure	10 mg BID (81%); 5 mg BID (14%); 11 mg QD (5%)	Week 12: response 42.9%, remission 33.3%; week 52: remission 41.2%
Latin America, 2024 [48]	Tofacitinib	Case series; *n* = 4; median age 14.5 y; UC	5 mg BID	Week 8: 3/4 remission; sustained 6–12 mon in 2 cases
Ireland, 2023 [49]	Tofacitinib	Single-center retrospective; *n* = 15; mean age 12.9 y; UC; anti-TNF failure	10 mg BID; average 0.39 mg/kg/dose	PUCAI decreased from 48.7 to 16.7; 8/15 remission at week 8
International, 2025 [50]	Upadacitinib	Multicenter retrospective; *n* = 100; median age 15.6 y; UC/IBD-U; biologic-experienced	45 mg/d (87%); weight-based < 40 kg; tapered to 30 mg/d	Week 8: SF-CR 56%, remission 62%, response 84%; PUCAI decrease from 45 to 0
International, 2025 [51]	Upadacitinib	Multicenter retrospective; *n* = 100; median age 15.8 y; active CD; biologic-experienced	45 mg/d; < 40 kg: 1.2–1.3 mg/kg/d	Week 8: response 75%, remission 56%, SF-CR 52%; > 50% CRP and FC normalization
USA, 2023 [52]	Upadacitinib	Case report; 12-y-old girl; non-stricturing CD; therapy failures	45 mg/d → 15 mg/d	PCDAI 0 at week 4; steroid-free remission at week 8
USA, 2024 [53]	Upadacitinib	Case series; *n* = 3; adolescents with ASUC; IFX + IV steroid failure	45 mg/d induction	PUCAI improved > 20 points in all; 1 remission; 1 colectomy; 1 switched to VDZ
China, 2024 [54]	Upadacitinib	Case report; 11-y-old boy; CD with ATM mutation; growth delay	45 mg/d → 15 mg/d	PCDAI decreased to 5 at week 8; SES-CD decreased from 27 to 12; BMI increased
China, 2025 [55]	Upadacitinib	Single-center retrospective; *n* = 8; median age 10.7 y; UC (6), CD (2)	45 mg/d (62.5%); 30 mg/d (37.5%); 75% + ustekinumab	UC: 33.3% endoscopic remission (24 wk); CD: 100% response
USA, 2024 [56]	Upadacitinib	Case report; 13-y-old girl; UC; 6-y disease; 5 prior failures	45 mg/d → 30 mg/d	Rapid clinical and FC response; sustained remission 9 mon
USA, 2025 [57]	Upadacitinib	Single-center retrospective; *n* = 20; median age 15 y; CD/UC/IBD-U; multi-failure	45 mg/d (75%); 30–15 mg/d; some combo regimens	Week 12 SF-CR 75% (CD 44%, UC/IBD-U 100%); CRP normalized 80%; IUS remission 60%
USA, 2024 [58]	Upadacitinib	Single-center retrospective; *n* = 20; median age 16.3 y; UC/IBD-U; biologic-experienced	45 mg/d induction; 30 mg/d maintenance	Week 8–12: response 90%, SF-CR 75%; week 24: SF-CR 65%
USA, 2025 [59]	Upadacitinib	Case series; *n* = 5; adolescents (14–18 y) with ASUC; anti-TNF refractory	30 mg BID intensified dosing	All colectomy-free during follow-up (55–203 d); 60% in steroid-free clinical/biochemical remission; 3/5 required dual therapy (UPA + UST)
USA, 2022 [60]	Ruxolitinib	Case series; *n* = 6; VEO-IBD; 50% onset < 1 y	Average 5.6 mg/m^2^ BID; + IL-1RA or anti-TNF	Response within 1 wk; at 6 mon: EIM resolution, TPN weaned, mucosal healing 3/6
International, 2020 [61]	Ruxolitinib	Case report; 7-mon-old girl; STAT1 GOF mutation	5–15 mg/m^2^ BID	Partial improvement; diarrhea/TPN dependence recurred

All studies are original publications; no copyright permission required. *UC* ulcerative colitis, *CD* Crohn’s disease, *IBD-U* inflammatory bowel disease unclassified, *VEO-IBD* very-early-onset inflammatory bowel disease, *ASUC* acute severe ulcerative colitis, *BID* twice daily, *QD* once daily, *TID* three times daily, *SF-CR* steroid-free clinical remission, *PUCAI* pediatric ulcerative colitis activity index, *PCDAI* pediatric Crohn’s disease activity index, *CRP* C-reactive protein, *FC* fecal calprotectin, *IUS* intestinal ultrasound, *SES-CD* simple endoscopic score for Crohn’s disease, *TPN* total parenteral nutrition, *EIM* extraintestinal manifestation, *VDZ* vedolizumab, *IL-1RA* interleukin-1 receptor antagonist, *GOF* gain-of-function, *UST* ustekinumab

#### JAK inhibitors

Tofacitinib, a JAK1/3 inhibitor, is the only JAK inhibitor with publicly available usage data for pIBD. A multicenter retrospective study of 101 children with moderate to severe UC reported an 8-week clinical response rate of 30%, with a steroid-free remission rate of 16%. Some patients managed to maintain remission for up to 24 weeks, resulting in significant reductions in fecal calprotectin levels [41]. Additional case series further indicate that tofacitinib can induce rapid remission in children who have failed multiple biologic therapies, with some avoiding colectomy and achieving histological improvements lasting over 6 months [42–49]. A prospective multicenter trial (NCT04624230) is currently evaluating the efficacy and safety of tofacitinib in children aged 2–17 years.

Upadacitinib, a selective JAK1 inhibitor, has shown promising results in several real-world studies. In a multicenter retrospective study of 100 children with unclassified UC or IBD, the 8-week steroid-free remission rate was 56%, with an overall clinical response rate of 84% [50]. In pediatric CD patients, upadacitinib was associated with a 75% response rate and a 52% steroid-free remission rate in patients who had previously failed multiple biologic therapies, along with significant improvements in inflammatory markers such as C-reactive protein and fecal calprotectin [51]. Additional case series further support the efficacy of upadacitinib in steroid-dependent and biologic-refractory patients. Some children have achieved rapid remission and maintained long-term steroid withdrawal. More recently, studies have shown its colectomy-sparing potential in adolescents with acute severe UC under intensified dosing regimens [52–59].

Moreover, ruxolitinib has shown potential value in pediatric patients with very early-onset IBD, particularly those with STAT1 gain-of-function mutations or immune deficiencies [60]. However, no registered clinical trials have been conducted with this drug, and its efficacy is limited by individual immune profiles. Currently, there are insufficient data regarding the use of baricitinib and filgotinib in pIBD, indicating the need for further clinical studies.

#### S1P modulators

The S1P signaling pathway regulates lymphocyte migration and immune homing through S1PR1–5 receptors, playing a critical role in regulating inflammation. Ozanimod, an oral S1PR1 and 5 selective modulators, has been approved for the treatment of moderate to severe UC in adults. Two phase II/III clinical trials are currently assessing its pharmacokinetics, efficacy, and safety in pediatric patients with moderate to severe UC (NCT05076175) and CD (NCT05470985). Additionally, etrasimod, another S1P modulator, is being studied in a phase II open-label trial in adolescents aged 12–17 years with moderate to severe UC (NCT05287126) to explore its preliminary efficacy and tolerability (Table 2). Notably, S1P plays a significant role not only in immune regulation but also in the development of the central nervous system and thymic T-cell maturation in children. While drugs such as ozanimod have shown therapeutic benefits in pediatric multiple sclerosis patients, the long-term safety of such treatments in pIBD patients must be carefully evaluated with respect to child development. Future studies should systematically clarify the therapeutic indications and long-term safety profiles of these S1P modulators in pIBD.

**Table 2:** Ongoing and recent clinical trials of small molecules in pediatric inflammatory bowel disease

Study (ID, year)	Drug	Phase	Population (age)	Disease	Sample size	Primary endpoint	Status
NCT04624230 (A3921210), 2021	Tofacitinib	III	Children 2–17 y	UC, moderate–severe	120	Clinical remission (Mayo score)	Recruiting (est. complete 2029)
NCT06332534 (U-EMPOWER), 2025	Upadacitinib	III	Children 2–18 y	CD, moderate–severe	110	Safety, efficacy, PK	Recruiting (est. complete 2031)
NCT05782907 (U-ASTOUND), 2025	Upadacitinib	III	Children 2–18 y	UC, moderate–severe	110	Safety, efficacy, PK	Recruiting (est. complete 2031)
NCT05076175 (IM047-001), 2022	Ozanimod	II/III	Children 2–17 y	UC, moderate–severe	120	Remission; safety, PK/PD	Recruiting (est. complete 2031)
NCT05470985 (IM047-023), 2023	Ozanimod	II/III	Children 2–17 y	CD, moderate–severe	5	PCDAI < 10; SES-CD ≤ 2–4	Completed, results pending
NCT05287126 (APD334-207), 2022	Etrasimod	II	Adolescents 12–17 y	UC, moderate–severe	36	Safety, efficacy, PK	Recruiting

Data source: ClinicalTrials.gov (accessed May 1, 2025). All data derived from public registry; no copyright permission required. *UC* ulcerative colitis, *CD* Crohn’s disease, *PK* pharmacokinetics, *PD* pharmacodynamics, *PCDAI* pediatric Crohn’s disease activity index, *SES-CD* simple endoscopic score for Crohn’s disease

### Special challenges in pediatric populations

Small-molecule targeted therapies are becoming increasingly integral components of treatment strategies for pIBD. However, balancing efficacy with safety remains a major challenge. Real-world studies have reported adverse events with JAK inhibitors in children, including herpes zoster and mild cytopenia with tofacitinib [41], as well as hyperlipidemia, acne, and infections with upadacitinib. In rare cases, more severe complications have been described, such as neuroendocrine tumors, cytomegalovirus colitis, and deep vein thrombosis associated with concomitant oral contraceptive use [53, 58].

Notably, the ORAL surveillance trial in rheumatoid arthritis patients demonstrated increased risks of malignancy and major cardiovascular events with tofacitinib [62]. Such signals have not been reported in pIBD to date. Likewise, long-term extension studies in other pediatric conditions, including juvenile idiopathic arthritis and atopic dermatitis, have not identified safety concerns related to malignancy or major cardiovascular events [63, 64]. Even so, these potential risks cannot be dismissed, particularly given the likelihood of prolonged exposure in pediatric patients, underscoring the importance of systematic long-term safety monitoring in pIBD. For clarity, adverse events are summarized by age group in Table 3, without confirming age-dependent differences.

**Table 3:** Reported adverse events of small-molecule therapies in pediatric inflammatory bowel disease

Group	Drug(s)	Reported adverse events	References
VEO-IBD (< 6 y)	Ruxolitinib	Oral HSV (case); *Clostridioides difficile* infection (1), otitis media (2), no cytopenias (cohort)	[60, 61]
Children (6–11 y)	Tofacitinib	None (9 y case)	[46]
	Upadacitinib	Mild leukopenia (11 y case); HZV (1, recovered); thrombocytopenia (1)	[54, 55]
Adolescents (12–18 y)	Tofacitinib	HZV (2), mild cytopenias (2), herpetic pharyngitis (1), postoperative obstruction (1), HLD (1), transient HLD + leukopenia (1), aseptic peritonitis (1)	[41–45, 47–49]
	Upadacitinib	Acne (including mild cases and 2/5 cases resolved with topical therapy), HLD, infections, lymphopenia, increased ALT, CMV colitis (1), *Clostridioides difficile* + surgery (1), DVT (OCP-related, 1), neuroendocrine tumor (1)	[50–53, 56–59]

Adverse events are summarized as reported in individual studies; numbers in parentheses indicate the number of cases when specified. All studies are original publications; no copyright permission required. *VEO-IBD* very-early-onset inflammatory bowel disease, *HZV* herpes zoster virus, *HSV* herpes simplex virus, *HLD* hyperlipidemia, *ALT* alanine aminotransferase, *CMV* cytomegalovirus, *DVT* deep vein thrombosis, *OCP* oral contraceptive

Children may be particularly vulnerable at certain developmental stages. The JAK/STAT pathway plays an essential role not only in immune regulation but also in bone metabolism and growth plate function. Experimental and clinical studies have shown that STAT3 activity in growth plate chondrocytes promotes differentiation and bone lengthening, while JAK1/STAT3 signaling in osteoblasts and osteocytes supports bone formation and remodeling; disruption of this pathway impairs skeletal development [65]. Therefore, prolonged pharmacologic inhibition raises concerns regarding growth. Similarly, the S1P signaling axis contributes to myelination and brain development, yet safety data on S1P inhibitors in infants and pre-adolescents are limited [66, 67]. In the PARADIGMS trial of pediatric multiple sclerosis, fingolimod reduced relapse rates. However, it was also associated with higher rates of infections and seizures [68], raising concerns about potential risks in younger populations.

Given that children’s immune systems are still developing, they may be especially susceptible to the effects of immunosuppressive therapy. Extended use could increase infection risk, blunt vaccine responses, and interfere with immune tolerance. Herpes zoster, which occurs in a dose-dependent manner in adults, has also been reported in pediatric patients, both as initial episodes and recurrences, while lymphocytopenia may have implications for vaccine efficacy and long-term immune reconstitution [41]. Additionally, the immaturity of the cytochrome P450 enzyme system and renal function poses risks when adult dosages are extrapolated, and the lack of age-appropriate liquid formulations further complicates dosing and adherence [69].

In conclusion, the use of small-molecule therapies in pIBD requires careful consideration of developmental stage, immune maturity, and metabolic capacity. Personalized, developmentally sensitive risk assessments and dosing strategies should be prioritized. Future studies must integrate pediatric pharmacology, developmental immunology, and real-world data to establish robust long-term safety frameworks.

### Future directions

The clinical application of small-molecule therapies in pIBD is still limited because of a lack of robust clinical evidence, significant differences in pharmacokinetics, and insufficient long-term safety data. Although drugs, such as tofacitinib, upadacitinib, ruxolitinib, ozanimod, and etrasimod, have shown promising efficacy in some pediatric patients, their off-label use and safety monitoring rely on adult clinical data and experience. To improve therapeutic precision and safety for children, future research needs to systematically explore developmental differences in drug response, molecular target variability, and the unique characteristics of the pediatric immune system.

Children’s gastrointestinal and immune systems are still maturing, which leads to significant differences from adult physiology, particularly in immune cell composition, target expression profiles, and drug metabolism. These developmental differences require the design of studies that are stratified by age and consider key physiological and immunological milestones. For example, treatment protocols and inclusion criteria should align with developmental windows, such as early childhood and adolescence, to better define the risk‒benefit profile of small-molecule therapies. Single-cell and spatial transcriptomic technologies offer valuable tools for exploring the dynamic states of immune cells, the spatial distribution of therapeutic targets, and context-dependent activation of signaling pathways. Integrating these high-resolution atlases across different pediatric age groups could help reveal age-related mechanisms of treatment response and identify novel, developmentally regulated therapeutic targets.

Additionally, the unique physiological characteristics of children, such as underdeveloped hepatic enzyme systems and immature intestinal barriers, limit the effectiveness of traditional pharmacokinetic models. Physiologically based pharmacokinetic modeling, which incorporates age-specific anatomical, physiological, and enzymatic factors, offers a more accurate way to predict drug absorption, distribution, metabolism, and excretion in children. This approach can inform more rational dosing strategies and help predict potential toxicity risks [70]. However, the use of physiologically based pharmacokinetic models in pIBD is still in its infancy, highlighting the need for more pediatric-specific datasets and well-validated models to support clinical applications.

Moreover, the immunopathological complexity of pIBD further complicates the prediction of disease progression and therapeutic response. Common biomarkers such as C-reactive protein and fecal calprotectin often lack sufficient sensitivity and specificity in children. To overcome these limitations, future research should focus on integrating multi-omics data within a developmental context to identify age-related molecular and cellular biomarkers. These biomarkers could improve disease monitoring, help predict therapeutic responses earlier, and offer insights into long-term prognosis.

Finally, the design of clinical trials for pIBD patients presents distinct challenges. Ethical considerations limit the use of invasive procedures and long-term placebo exposure. Recruitment is often slow, given the relatively small patient pool and the hesitancy of families to participate in experimental protocols. Careful age stratification is also necessary, since the pharmacology and outcomes of therapy may differ between a young child and an older adolescent. These challenges partly explain the scarcity of large-scale pediatric trials and emphasize the importance of collaborative, multicenter efforts to generate data that can truly guide clinical practice. Recently, the IBD Porto Group published a consensus response to the FDA draft guidance on pIBD drug approval trials, underscoring the urgent need for innovative, ethically sound, and developmentally tailored trial designs to accelerate the development and approval of novel therapies in children [71].

## Conclusions

The integration of developmental biology, advanced omics technologies, and pediatric-specific pharmacological models is crucial for advancing precision medicine in pIBD. Moving away from adult-derived treatment paradigms toward a framework that is informed by developmental stages and underlying mechanisms will be essential to optimizing the safety and efficacy of small-molecule therapies for children.

## Data Availability

This article is a review and does not report any new data. All data supporting the findings of this study are available in the published literature cited herein.
